# High production of fatty alcohols in *Escherichia coli* with fatty acid starvation

**DOI:** 10.1186/s12934-016-0524-5

**Published:** 2016-07-27

**Authors:** Yilan Liu, Sha Chen, Jinjin Chen, Jiemin Zhou, Yanyan Wang, Maohua Yang, Xianni Qi, Jianmin Xing, Qinhong Wang, Yanhe Ma

**Affiliations:** 1 Tianjin Institute of Industrial Biotechnology, Chinese Academy of Sciences, 32 XiQiDao, Tianjin Airport Economic Area, Tianjin, 300308 China; 2 Institute of Chinese Materia Medica, China Academy of Chinese Medical Sciences, No. 16, Nanxiaojie, Dongzhimennei, Beijing, 100700 China; 3 State Key Laboratory of Biochemical Engineering, Institute of Process Engineering, Chinese Academy of Sciences, Beijing, 100190 China

**Keywords:** *Escherichia coli*, Fatty alcohols, Acyl-ACP thioesterases, Fatty acid starvation

## Abstract

**Background:**

Microbial biofuel synthesis attracting increasing attention. Great advances have been made in producing fatty alcohols from fatty acyl-CoAs and fatty acids in *Escherichia coli*. However, the low titers and limited knowledge regarding the basic characteristics of fatty alcohols, such as location and toxicity, have hampered large-scale industrialization. Further research is still needed.

**Results:**

In this study, we designed a novel and efficient strategy to enhance fatty alcohol production by inducing fatty acid starvation. We report the first use of deletions of acyl-ACP thioesterases to enhance fatty alcohol production. Transcriptional analysis was conducted to investigate the mechanism of the designed strategy. Then, fatty alcohol production was further enhanced by deletion of genes from competing pathways. Fatty alcohols were shown to be extracellular products with low toxicity. The final strain, *E. coli* MGL2, produced fatty alcohols at the remarkable level of 6.33 g/L under fed-batch fermentation, representing the highest reported titer of fatty alcohols produced by microorganisms.

**Conclusions:**

Deletions of genes responsible for synthesis of fatty acids and competing products are promising strategies for fatty alcohol production. Our investigation of the location and toxicity of fatty alcohols suggest bright future for fatty alcohol production in *E. coli*.

**Electronic supplementary material:**

The online version of this article (doi:10.1186/s12934-016-0524-5) contains supplementary material, which is available to authorized users.

## Background

The increasing demand and limited supply of fuels has given rise to concern regarding prospects for sustainable development [1, 2]. Microbial production of high-energy fuels has emerged as a viable alternative to conventional fuels [3, 4]. Fatty acids and their derivatives are of particular interest owing to their high caloric value [5]. *Escherichia coli* is suitable for this purpose, not only because fatty acid metabolism in *E. coli* is well understand [6] but also because genetic techniques for *E. coli* have been extensively investigated [7, 8]. In the past decade, significant efforts have been made to produce fatty acids, alcohols and alkanes in *E. coli* [5, 9–13]. Among those chemicals, fatty alcohols have attracted increasing attention because they can be widely used in medicines, cosmetics, detergents and skin care products [14].

Fatty alcohols can be produced from fatty acyl-ACPs, fatty acyl-CoAs, or fatty acids through the catalysis of fatty acyl reductase (*FAR*) [12, 15–17]. Great advances have been made in engineering microorganisms to produce fatty alcohols from fatty acyl-CoAs [5, 16] and fatty acids [17]. Three main strategies have been applied in these studies. First, a variety of fatty acyl reductases from marine bacteria, soil bacteria and plants have been expressed in *E. coli* to facilitate fatty alcohol production [16–19]. Second, genes related to fatty alcohol synthesis have been overexpressed [20]. Third, genes responsible for fatty alcohol degradation have been knocked out [18]. However, the highest reported titers of even and odd-chain fatty alcohols are only 3.78 and 1.9 g/L, respectively [20, 21], still far below levels suitable for industrialization.

In this study, a novel strategy was developed to enhance fatty alcohol production by inducing fatty acid starvation. Cellular location and toxicity studies of fatty alcohols are a crucial step that must occur before future industrialization. However, to our knowledge, no previous research has addressed these issues performed on these. Therefore, investigations on toxicity and the cellular localization of fatty alcohols were conducted.

## Results and discussion

### Enhancing fatty alcohol production via inducing fatty acid starvation

**Fig. 1:**
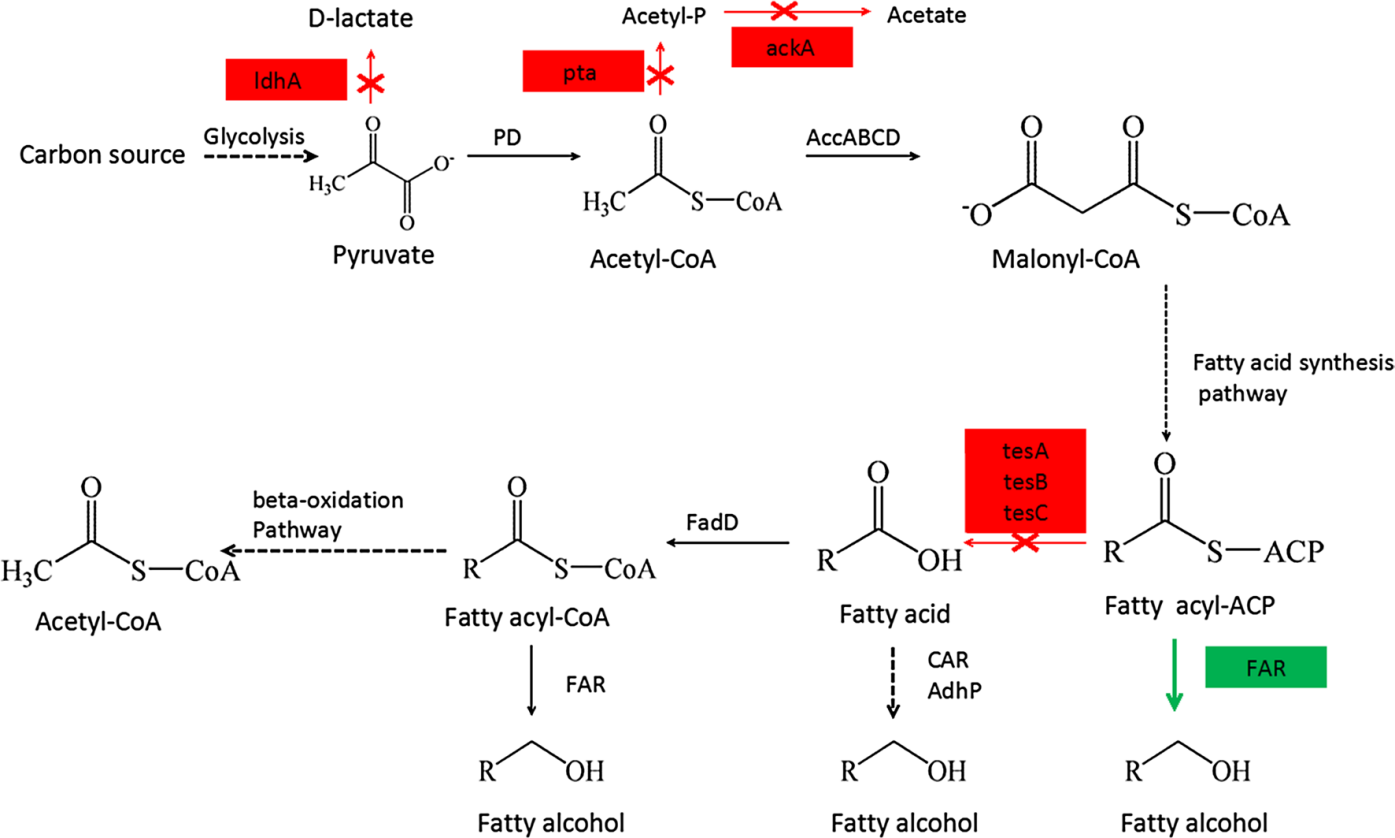
Engineered pathway for fatty alcohol production in *E. coli*. The fatty alcohol synthesis flux from fatty acyl-ACP was enhanced by deletion of acyl-CoA thioesterases coupled with overexpression of fatty acyl-ACP reductase (*FAR*, *green line*). Competing pathways were blocked (*red line*) by deletion of lactic dehydrogenase (*ldhA*), phosphate acetyltransferase (*pta*) and acetate kinase (*ackA*)

**Fig. 2:**
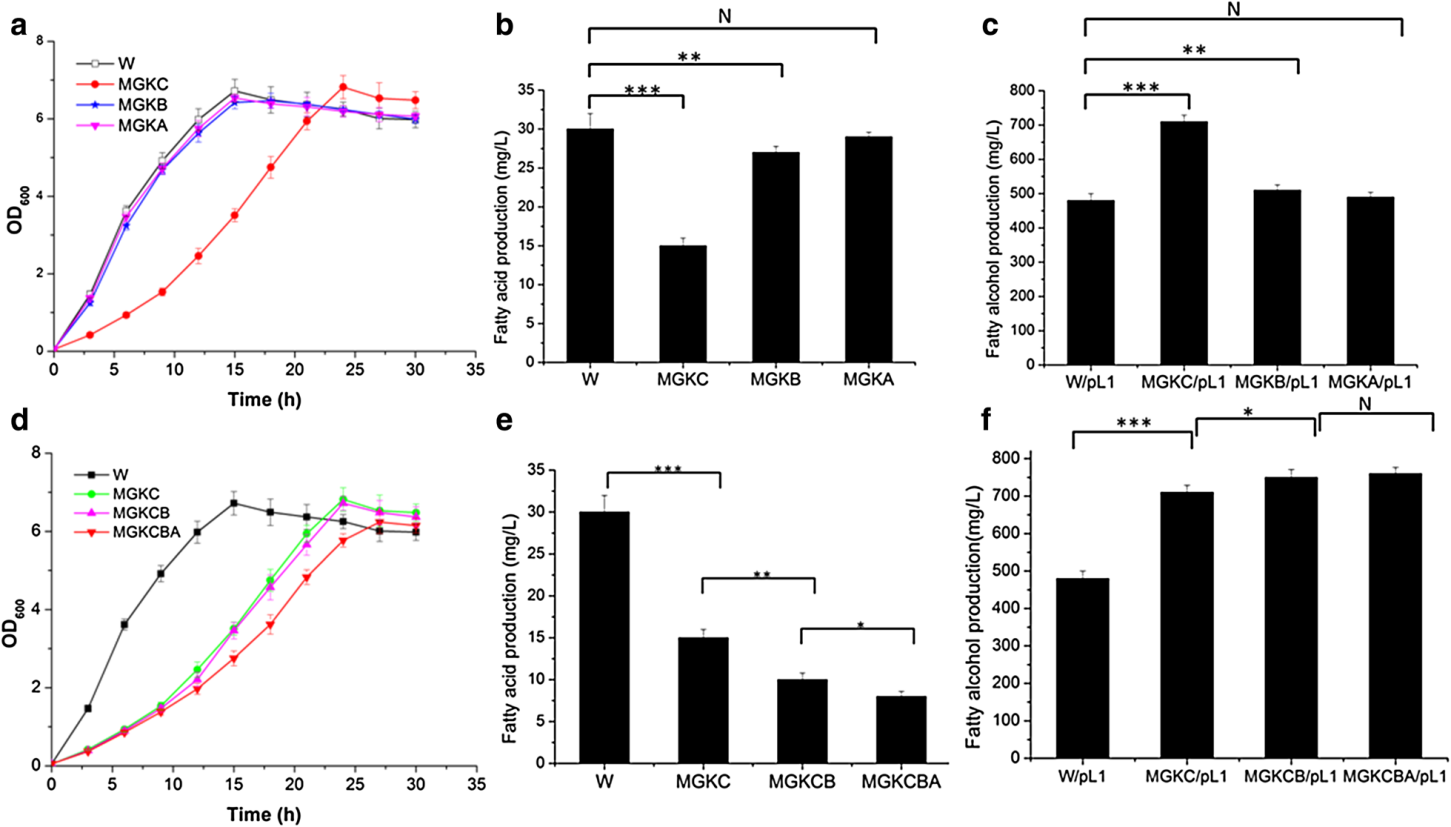
Fatty alcohol and fatty acid accumulation in engineered strains. The values are the mean of three biological replicates ± standard deviations (n = 3). **a**–**c** The growth curves and fatty acid and fatty alcohol production levels in single acyl-ACP thioesterase deletion strains. **d**–**f** The growth curves, fatty acid production and fatty alcohol production levels in multiple acyl-ACP thioesterase deletion strains. (***) p < 0.001, (**) p < 0.01, (*) p < 0.05, (N) p > 0.05, one-way ANOVA. For fatty acid and fatty alcohol production, data were observed 24 h after inoculation in all strains except for MGKCBA/pL1, in which the data were observed 36 h after inoculation

Fatty acids are a crucial component of all living organisms [22]. If the concentration of fatty acids drops, resulting in fatty acid starvation, the expression levels of genes for fatty acid synthesis are upregulated to satisfy growth needs. This process eventually leads to the accumulation of fatty acyl-ACPs, which are reduced to fatty alcohols by *FAR* (Fig. 1). There are various ways to block fatty acid formation, such as the mutation of *accD* (a subunit of acetyl-CoA carboxylase) [23]. In this study, acyl-ACP thioesterases rather than upstream genes were deleted to block fatty acid formation and to enhance fatty alcohol production (Fig. 1). There are three reported acyl-ACP thioesterases in *E. coli: tesA*, *tesB* [24] and *tesC* [25]. In a subcellular localization analysis using Cell-PLoc 2.0, *tesA*, *tesB* and *tesC* were predicted to be located in the periplasm, inner membrane and cytoplasm, and cytoplasm, respectively [26]. Because fatty acids are synthesized in the cytoplasm in *E. coli*, *tesC* may play a key role in producing fatty acids. Moreover, it has been reported that the Michaelis constants (km) of *tesA* and *tesB* for native palmitoyl-ACP are 100 to 200 pM, which are over more than tenfold higher than those for palmitoyl-CoA [24]. These finding sugggest that *tesA* and *tesB* do not play major roles in producing fatty acids. Therefore, deletions of *tesC*, *tesB* and *tesA* were performed individually (Table 1). The fermentation results supported our presumption that the deletion of *tesC* would dramatically affect growth rate, fatty acid production and fatty alcohol production, whereas the effects of *tesB* and *tesA* deletions were less significant (Fig. 2a–c).

**Table 1:** Plasmids and strains used in this study

Plasmids/strains	Relevant characteristic(s)	Reference/source
Plasmids		
pMD™ 18-T Vector	T-easy vector	Takara
pTrcHisA	Expression vector	Invitrogen
pKD46	Bla γβexo temperature conditional pSC101 replicon	[8]
pEASY-cat-sacB	T-easy vector with cat-sacB cassette	Lab collection
pL1	pTrcHisA containing the *M. aquaeolei FAR* gene	This study
Strains		
W	*E. coli* K-12 MG1655	Lab collection
MGKC	*E. coli* K-12 MG1655 Δ*tesC*	This study
MGKB	*E. coli* K-12 MG1655 Δ*tesB*	This study
MGKA	*E. coli* K-12 MG1655 Δ*tesA*	This study
MGKCB	*E. coli* K-12 MG1655 Δ*tesC* Δ*tesB*	This study
MGKCBA	*E. coli* K-12 MG1655 Δ*tesC* Δ*tesB* Δ*tesA*	This study
MGL1	*E. coli* K-12 MG1655 Δ*tesC* Δ*tesB* Δ*ldhA::kan* Δ*pta* Δ*ackA*	This study
W/pL1	W bearing pL1	This study
MGKC/pL1	MGKC bearing pL1	This study
MGKB/pL1	MGKB bearing pL1	This study
MGKA/pL1	MGKA bearing pL1	This study
MGKCB/pL1	MGKCB bearing pL1	This study
MGKCBA/pL1	MGKCBA bearing pL1	This study
MGL2	MGL1 bearing pL1	This study

Subsequently, *tesB* and *tesA* were deleted sequentially from the MGKC strain to enhance fatty alcohol production. The growth curves of the engineered strains (MGKC, MGKCB and MGKCBA) are shown in Fig. 2d. Interestingly, although the growth rates decreased after thioesterase deletions, the final cell densities of the engineered strains were similar. Beyond our expectations, after all three yet known fatty acyl-ACP thioesterases were knocked out, the *E. coli* still survived. Other thioesterases in *E. coli*, such as *ybhC* (acyl-CoA thioesterase) and *paaI* (hydroxyphenylacetyl-CoA thioesterase), might possibly serve anaplerotic functions. The amounts of fatty alcohols and fatty acids produced in wild-type and engineered strains with *FAR* expression (W/pL1, MGKC/pL1, MGKCB/pL1 and MGKCBA/pL1) are shown in Fig. 2e, f. W represents the *E. coli* K-12 MG1655 wild-type strain in this study. The highest production of fatty alcohols and fatty acids in strains W/pL1, MGKC/pL1 and MGKCB/pL1 was observed 24 h after inoculation, whereas for strain MGKCBA/pL1, production peaked after 36 h. The fatty alcohol titer increased from 480 to 710 mg/L as a result of the deletion of *tesC,* whereas, fatty acid production decreased from 30 to 15 mg/L. Similarly, the fatty alcohol titer increased to 750 mg/L as a result of the subsequent deletion of *tesB* whereas, fatty acid production decreased to 10 mg/L (Fig. 2). As a result of the subsequent *tesA* deletion, the fatty acid titer decreased to 8 mg/L, whereas the fatty alcohol titer increased to 760 mg/L. Overall, the results demonstrated that the strategy for enhancing fatty alcohol production by inducing fatty acid starvation was effective. Furthermore, the subcellular localization of *tesC* and the fermentation results suggest that *tesC* may play the key role in fatty acid formation in *E. coli*.

### Investigating the mechanism of fatty acid starvation by whole-genome transcriptional analysis

To explore the mechanism of enhancing fatty alcohol production with fatty acid starvation, whole-genome transcriptional analysis was performed. Sequence data are available in public databases (NCBI SRA accessions SRA200924). Strains MGKCBA/pL1 and W/pL1 were cultured and collected 12 h after isopropyl β-D-1-thiogalactopyranoside (IPTG) induction. The transcription levels of 44 genes in four modules closely related to fatty alcohol production changed (Table 2). (a) The transcription levels of most genes from the glycolysis module were upregulated, particularly *ptsG* (3.37-fold, p = 0.0023), thus implying that the engineered strain accelerated glycolysis and therefore supplied more precursors for fatty alcohol synthesis (Fig. 1). (b) The transcription levels of most genes from the TCA cycle (except for *acnA*) were downregulated, particularly *sdhA* (0.29-fold, p = 0.0031), thus implying that the engineered strain reduced the consumption of the carbon source for cell growth, and this surplus carbon source could then be used for fatty alcohol production. (c) The transcription levels of most genes from the fatty acid synthesis module were upregulated, thereby satisfying the growth demands. (d) The transcription levels of most genes from the fatty acid degradation module were downregulated, particularly *fadB* (0.36 fold, p = 0.0263), thus implying that the fatty acids produced by the engineered strain were mainly used to satisfy growth needs, hence, fewer fatty acids were degraded. Moreover, no reads from *tesA*, *tesB* or *tesC* were observed in strain MGKCBA/pL1, thus indicating that these genes were successfully deleted. Additionally, the most highly upregulated gene was *ybbO* (4.12 fold, p = 0.0131), an NADP^+^-dependent aldehyde reductase that oxidizes alcohol to its corresponding aldehyde. This gene may have assisted in the synthesis of fatty acids from fatty alcohols and partially satisfied the growth need partially. Aside from the deleted genes, the most highly down regulated gene was *yibG* (0.162 fold, p = 0.0265). However, *yibG’*s function is still unknown [27]. Cell-PLoc 2.0 predicted that the protein encoded by the *yibG* gene is located in cell inner membrane [26]. Blastp analysis of *yibG* (Domain Enhanced Lookup Time Accelerated BLAST, NCBI) suggested that it might be a tetratricopeptide-like repeat protein, moreover, this protein has been reported to be involved in the stress response via protein–protein interactions [28, 29]. Therefore, *yibG* downregulation may adapt the protein interaction in the *E. coli* inner membrane in response to the induced fatty acid starvation.

**Table 2:** Transcriptional analysis for genes from the glycometabolism and fatty acid pathway

Gene name	Fold change	*p* value	Description	Gene name	Fold change	*p* value	Description
*Crr*	1.19	0.1422	Glycolysis	*pcK*	0.75	0.4359	TCA
*ptsG*	3.37	0.0023	Glycolysis	*gltA*	0.38	0.0138	TCA
*Pgi*	2.00	0.0669	Glycolysis	*Icd*	0.58	0.1293	TCA
*pfkB*	2.17	0.0687	Glycolysis	*sucA*	0.49	0.0584	TCA
*pfkA*	1.52	0.2580	Glycolysis	*sucB*	0.45	0.0315	TCA
*fbaB*	2.72	0.0095	Glycolysis	*sucC*	0.83	0.6182	TCA
*fbaA*	1.49	0.3091	Glycolysis	*sucD*	0.83	0.6182	TCA
*gapA*	1.22	0.6181	Glycolysis	*sdhA*	0.29	0.0031	TCA
*ytjC*	1.13	0.8491	Glycolysis	*sdhB*	0.24	0.0012	TCA
*gpmA*	1.65	0.1889	Glycolysis	*sdhC*	0.83	0.0031	TCA
*Eno*	1.36	0.4461	Glycolysis	*sdhD*	0.83	0.0031	TCA
*pykF*	1.34	0.4338	Glycolysis	*fumA*	0.30	0.0020	TCA
*pykA*	1.42	0.3519	Glycolysis	*fumC*	0.66	0.2889	TCA
*acnA*	1.28	0.4994	TCA	*Mdh*	0.81	0.5553	TCA
*accA*	1.31	0.4684	Fatty acid synthesis	*Mqo*	0.64	0.2180	TCA
*accC*	1.48	0.3063	Fatty acid synthesis	*fadD*	0.66	0.2633	Fatty acid degradation
*accD*	1.39	0.3625	Fatty acid synthesis	*fadE*	0.58	0.1408	Fatty acid degradation
*fabD*	1.58	0.2078	Fatty acid synthesis	*fadB*	0.36	0.0263	Fatty acid degradation
*fabH*	1.58	0.2090	Fatty acid synthesis	*fadJ*	0.82	0.6126	Fatty acid degradation
*fabB*	1.17	0.6594	Fatty acid synthesis	*fadA*	0.54	0.1024	Fatty acid degradation
*fabG*	1.41	0.3477	Fatty acid synthesis	*fadI*	0.82	0.6126	Fatty acid degradation
*fabZ*	1.67	0.1716	Fatty acid synthesis	*yqeF*	0.37	0.0175	Fatty acid degradation

Genes with fold-change value >1.1 and <0.9 are shown

In summary, the deletion of acyl-ACP thioesterases resulted in the upregulation of expression of most genes associated with glycolysis and fatty acid synthesis. This upregulation led to the accumulation of fatty acyl-ACPs, which were finally reduced to fatty alcohols by the expressed *FAR* (Fig. 1). Furthermore, the downregulation of fatty acid degradation and the TCA module (Table 2) indicated that the designed strategy should be an economic choice for fatty alcohol production. Notably, the transcriptional upregulation of genes from the fatty acid synthesis module was less than 1.67-fold and did not show a statistically significant difference (Table 2). This behavior may be due to the stringent regulation of the fatty acid synthesis pathway. For example, it has been reported that *accB* acts as an autoregulator of the *accBC* operon [30], whereas *accAD* regulates its own translation by binding to the coding region of mRNA for both subunits [28]. Therefore, engineering efforts that focused on key enzymes may be helpful for the further enhancement of fatty alcohol production.

### Further improving fatty alcohol production by deleting genes from competing pathways

**Fig. 3:**
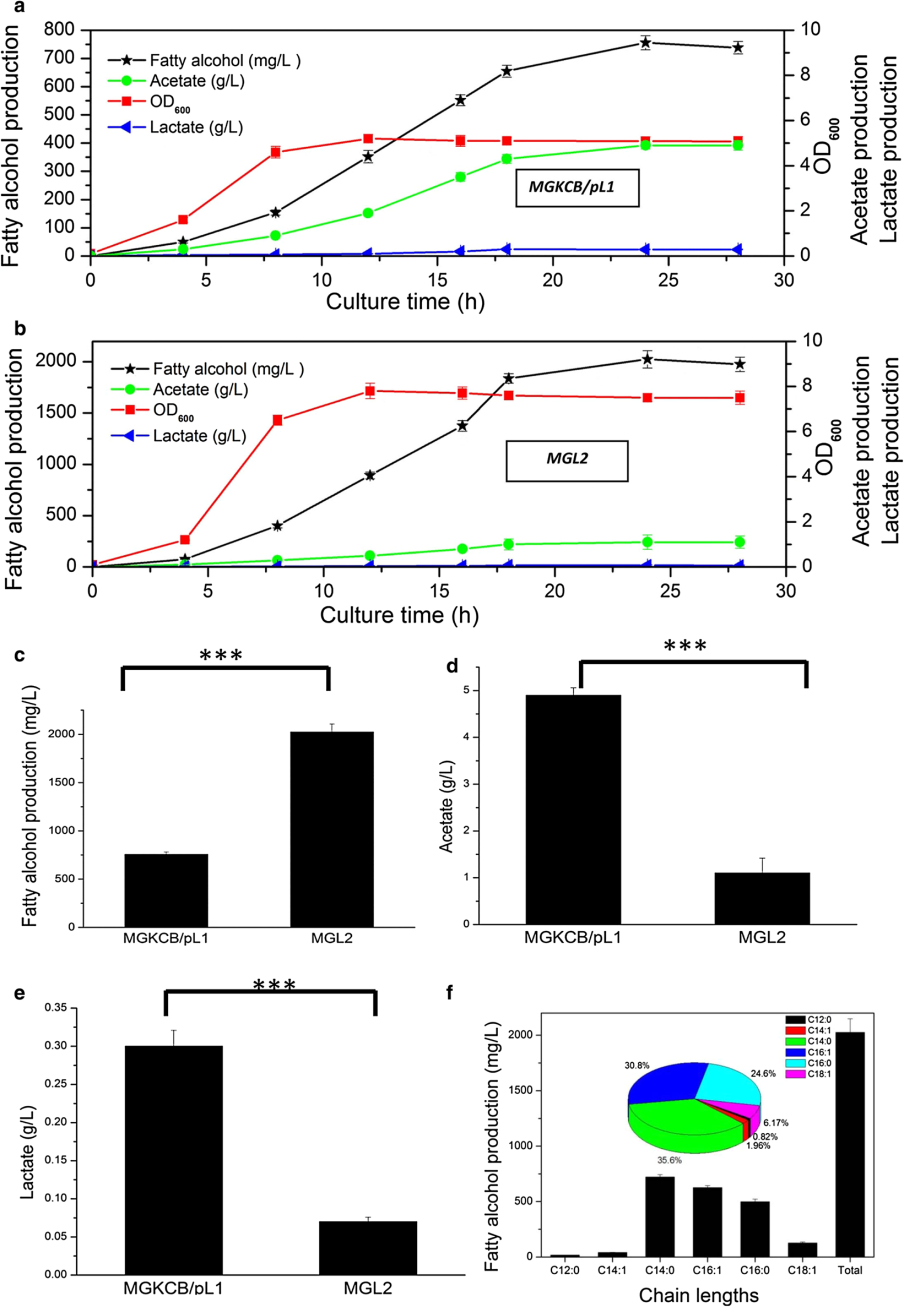
The effects of deleting genes from competing pathways on fermentation. **a** Fermentation results for MGKCB/pL1. **b** Fermentation results for MGL2. **c** Comparison of fatty alcohol production between MGKCB/pL1 and MGL2 strains. **d** Comparison of acetate accumulation between MGKCB/pL1 and MGL2 strains. **e** Comparison of lactate accumulation between MGKCB/pL1 and MGL2 strains. **f** Distribution of fatty alcohols with different carbon chain lengths in MGL2. The values are the means of three biological replicates. (***) p < 0.001, one-way ANOVA. **c**–**f**, data were observed 24 h after inoculation

The MGKCB strain was selected for the subsequent manipulation because it has much shorter fermentation period but a similar fatty alcohol production, as compared with MGKCBA/pL1 strain. To enhance fatty alcohol production, deletions of lactate dehydrogenase (*ldhA*), acetate kinase (*ackA*) and phosphate acetyltransferase (*pta*) were performed, resulting in the MGL2 strain. As shown in Fig. 3a, b, the highest optical density at 600 nm (OD_600_) was increased from 5.2 to 7.8. Moreover, the fatty alcohol titer increased from 756 to 2024 mg/L (Fig. 3a–c). The deletions of *ldhA*, *pta* and *ackA* dramatically decreased the production of lactate (from 0.1 to 0.06 g/L) and acetate (from 4.9 to 1.1 g/L) (Fig. 3d, e). Fatty alcohol productivity reached 259 mg/OD/L in the MGL2 strain, which is about approximately 1.7-fold higher than that of the MGKCB/pL1 strain.

The distribution of fatty alcohols produced by the MGL2 strain ranged from C12 to C18 (Fig. 3f), similarly to previously reported results [16]. MGL2 predominantly produced two saturated (C14:0 and C16:0) and two unsaturated (C16:1 and C18:1) fatty alcohols. The two major products, C14:0 and C16:1, accounted for 35.6 and 30.8 % of the total fatty alcohol production, respectively. Notably, this reported titer was achieved in MGL2 under shake flask fermentation for only 24 h. These results suggest that blocking fatty acid formation coupled with deleting competing pathways for acetyl-CoA pool efficiently enhanced fatty alcohol production.

### Fatty alcohol production by fed-batch fermentation

**Fig. 4:**
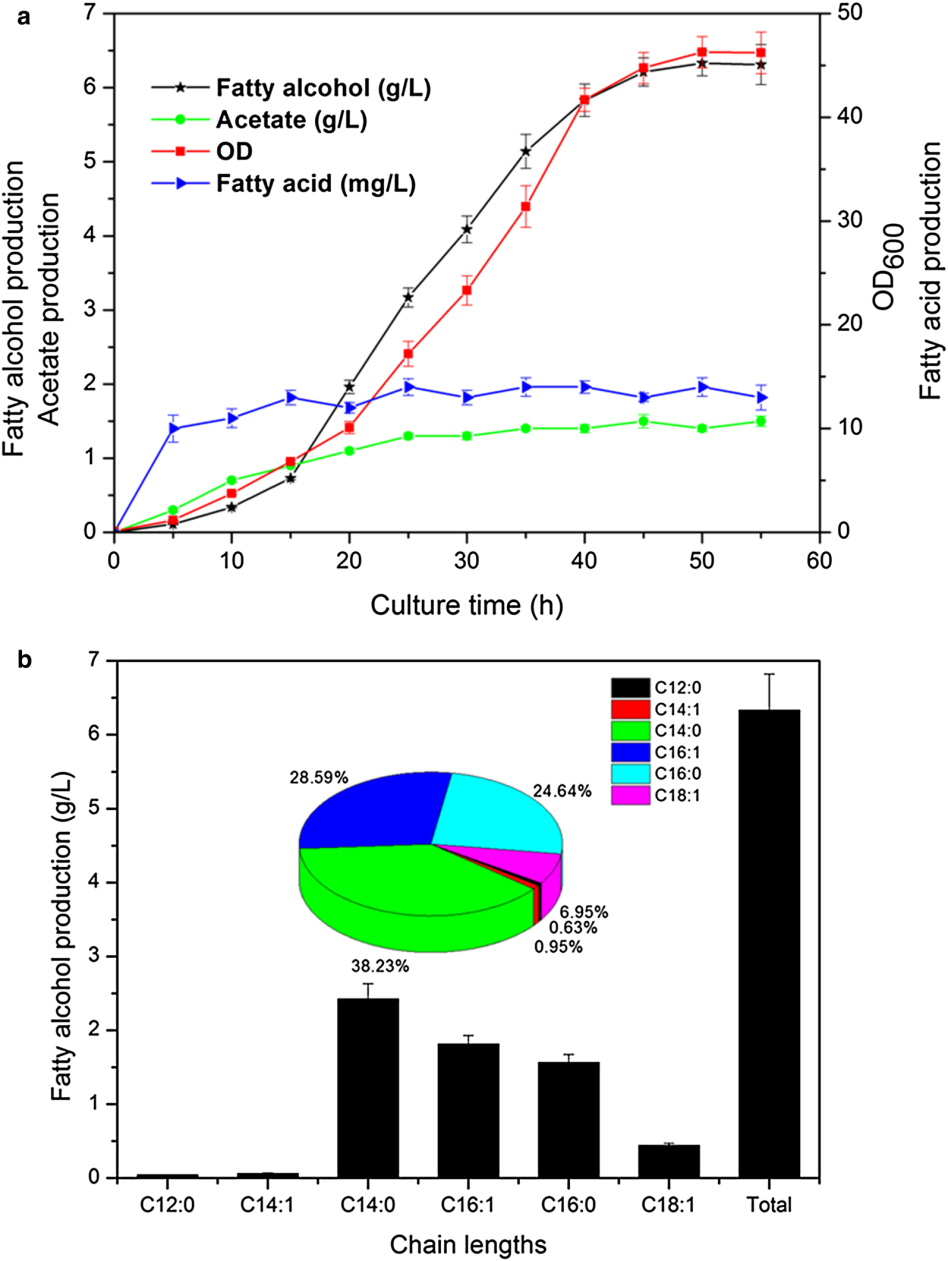
Results of fed-batch fermentation in MGL2. **a** Cell growth and fatty acid, fatty alcohol and acetate production levels. **b** Chain-length distribution of produced fatty alcohols

Fed-batch fermentations were performed using fermentation medium in 3-L Bioflo 110 with MGL2 strain. The total fatty alcohol accumulation reached a maximum titer of 6.33 g/L at 50 h, At which point, the OD_600_ reached 46 (Fig. 4a). Both the cell density and the fatty alcohol concentration stopped increasing after 50 h. Notably, the fatty acid concentration was lower than 12 mg/L during the entire fermentation process (Fig. 4a). Thus, the high fatty alcohol titer could be partly attributed to fatty acid starvation during fermentation. The fatty alcohol composition produced in MGL2 under fed-batch fermentation (Fig. 4b) was similar to that of shake-flask fermentation: two saturated (C14:0 and C16:0) and two unsaturated (C16:1 and C18:1) fatty alcohols were the major components. The percentage of unsaturated fatty alcohols reached up to 36.5 % of the total fatty alcohols (Fig. 4b). Additionally, the fed-batch fermentation with strain MGL2 was conducted in M9 medium with glycerol as the only carbon source. The final titer was 5.94 g/L (data not shown), which was also higher than any previously reported values. The results above demonstrated that our strategies might be useful in the industrial production of fatty alcohols.

### Investigation of the toxicity and cellular localization of fatty alcohols

**Fig. 5:**
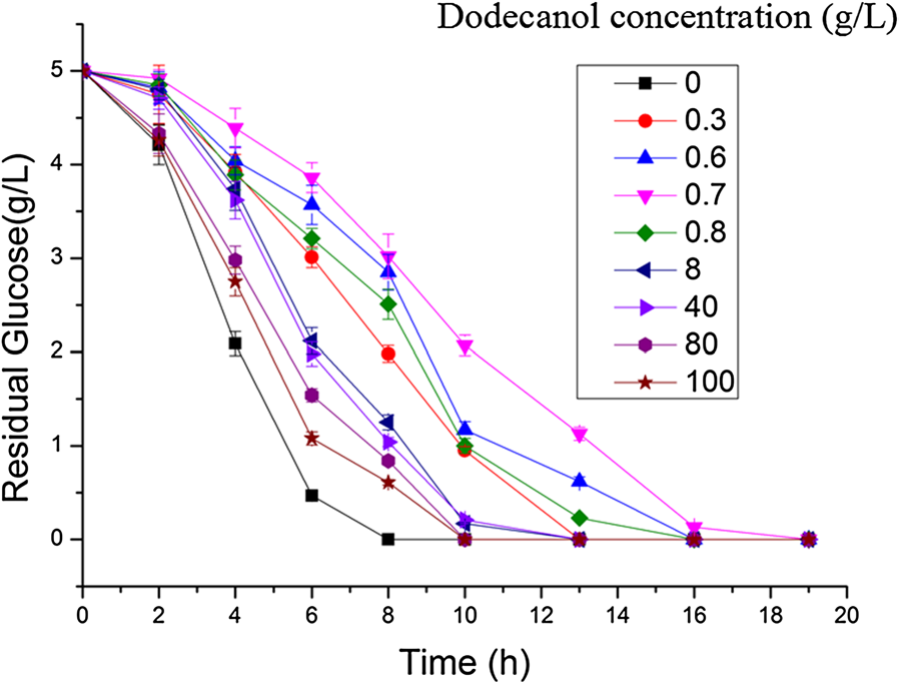
Dodecanol toxicity test. The wild-type *E. coli* MG1655 strain was cultured in LB medium with 5 g/L of glucose. Dodecanol was added in concentration ranging from 0 to 100 g/L. The values are the means of three biological replicates

Toxicity is vital for the microbial production of chemicals because physiological investigation has revealed a correlation between solvent toxicity to microbes and the logP value [31, 32], which has been defined as the partition coefficient of the given solvent in an equimolar mixture of octanol and water [33]. The lower the logP value, the higher the toxicity of the solvent [31]. Among the fatty alcohols produced, dodecanol have the lowest logP value and is supposed to have the highest toxicity. Hence, the toxicity of dodecanol to *E. coli* was analyzed. Figure 5 shows that, when the dodecanol concentration increased from 0 to 0.7 g/L, the toxicity increased simultaneously. As its concentration increased thereafter, the toxicity of dodecanol decreased, possibly because when the concentration was relatively low, the small droplets of dodecanol attached to the cell membranes and affected the mass transfer, thereby harming to cells. When the concentration became relatively higher, the small droplets of dodecanol might have adhered to each other instead, thus reducing the solvent’s toxicity to cells. These results suggested that overproduction of fatty alcohols in *E. coli* may be possible.

Cellular localization is also important for microbial productions. Intracellular products are limited by the maximum biomass, while the situation is different for extracellular products. The cellular localization of produced fatty alcohols was investigated via in situ product separation tests in the W/pL1 and MGL2 strains. As shown in Additional file 1: Table S1, the produced fatty alcohols were detected in the tridecanol layer, whereas, no fatty alcohol was detected in the medium or the cells. Thus, fatty alcohols produced in *E. coli* are extracellular products and will not be limited by the maximum biomass. Interestingly, the output of fatty alcohols in the in situ separation fermentation was slightly higher than that in the control, possibly because the produced fatty alcohols attached to cell membranes without the addition of extraction solvent, thus affecting mass transfer and fatty alcohol production. Notably, in the control test the fatty alcohols produced in strain W/pL1 adhered to cells after centrifugation, whereas the situation was different in MGL2. These results supported our speculation that fatty alcohol droplets at low concentration will attach tocells but instead attach to each other and are suspended in the medium after their concentration increase beyond a certain threshold.

## Conclusions

In summary, a fatty alcohol titer of 6.33 g/L was achieved in fed-batch fermentation in *E. coli* via the deletion of fatty acyl-CoA thioestarase, to starve cells of fatty acids, as well as deletion of *ldhA*, *pta* and *ackA* from competing pathways. Moreover, fatty alcohols were shown to be extracellular products with low toxicity to *E. coli*. These results indicate a bright future for microbial production of fatty alcohols.

## Methods

### Materials

Restriction enzymes and T4 ligase were purchased from Takara Biotechnology (Dalian, China). PCR purification kits, gel extraction kits, QIAprep Spin plasmid miniprep kits, were from Axygen (Union City, CA, USA). Oligonucleotide primers were synthesized by Sangon Biotechnology (Shanghai, China) (Additional file 1: Table S2). Fatty acid standards were purchased from Nu-Check-Prep (Elysian, MN, USA). Fatty alcohol standards were purchased from Dr. Ehrenstorfer GmbH (Augsburg, Germany).

### Bacterial strains and plasmid construction

All bacterial strains and plasmids used in this work are listed in Table 1. All deletions were performed with our recently developed method [8]. First, fragments containing *cat*-*sacB* flanked by tandem repeats were constructed with primers (Additional file 1: Table S2) and then introduced into the target site via intermolecular homologous recombination assisted by lambda red enzymes (pKD46). Then, recombinants were selected from Luria-Mertani (LB) agar plate containing chloramphenicol (34 mg/L). Seamless excision of the selectable marker was achieved using sucrose [8]. The *FAR* gene (maqu_2220) was amplified from *M. aquaeolei* VT8 DNA by PCR using the primers FARF and FARR. The PCR product was purified and digested with *BamH*I and *EcoR*I. After purification, the digested PCR fragment was cloned into the expression vector pTrcHisA, resulting in the recombinant plasmid pL1. *E. coli* MG1655 was used as the original strain.

### Culture conditions

LB medium was used to assess the effects of gene deletions on cell growth. To assess fatty alcohol production by different engineered *E. coli* strains, three single colonies of each strain were cultivated in LB medium containing 100 μg/mL ampicillin overnight at 37 °C. Each seed culture was inoculated into 50 mL of LB containing a mixed carbon source (0.5 % glucose, 2.5 % glycerol) in a 250-mL flask, and this was followed by incubation at 37 °C and 220 rpm. When the OD_600_ reached 0.6–0.8, IPTG was added to a final concentration of 0.2 mM. 5-mL cell culture was used for fatty alcohol analysis.

Fed-batch fermentation was performed with a medium volume of 1 L in a 3-L Bioflo 110 fermentor (New Brunswick Scientific, Edison, NJ, USA). Strains were cultivated in 40 mL of seed medium (5 g/L yeast extract, 10 g/L tryptone, 10 g/L glycerol) in a 250-mL flask containing 100 μg/mL of ampicillin overnight at 37 °C. The seed culture was inoculated into 1 L fermentation medium (10 g/L yeast extract, 20 g/L tryptone, 25 g/L glycerol, 3 g/L KH_2_PO4, 8 g/L Na_2_HPO_4_·12H_2_O, 0.2 g/L CaCl_2_, 100 μg/mL ampicillin, 1 mM MgSO_4_, 100 μg/mL ampicillin). When the OD_600_ reached 0.6–0.8, IPTG was added to a final concentration of 0.2 mM. The temperature was maintained at 37 °C, and the pH was kept at 7.00 by the addition of 25 % (v/v) NH_4_OH or 1 M HCl solutions. A constant glycerol feed-rate of 0.5 g/h was maintained. Agitation was provided by a single impeller with the stirring speed set between 200 and 500 rpm. The stirring speed was controlled to ensure that the dissolved oxygen (DO_2_) content was greater than 10 %. The air inflow rate was maintained at 3.0 L/min.

To assess the toxicity of fatty alcohol to *E. coli*, the original strain was cultured in LB medium containing 5 g/L glucose and dodecanol in different concentrations ranging from 0 to 100 g/L. Because the addition of dodecanol affects the quantitative measurement of biomass, the consumption of glucose and not biomass was analyzed to assess the toxicity. To investigate the location of produced fatty alcohols, in situ product separation tests were conducted in strains W/pL1 and MGL2. Colonies of each strain were cultured in LB medium containing 100 μg/mL of ampicillin overnight at 37 °C. The seed culture was inoculated into 50 mL of LB medium containing 2.5 % glycerol in a 250-mL flask at 37 °C and 220 rpm. When the OD_600_ reached 0.6–0.8, 0.2 mM IPTG was added. Tridecanol was used as an extraction solvent and added to the medium before inoculation. The solvent to sample ratio is 1:5. Medium without tridecanol was used as control.

### Analytic methods

The bacterial growth conditions were estimated from the OD_600_ of the medium with a spectrophotometer (723 N, Shanghai Precision & Scientific Instrument Co. Ltd, China). The concentrations of glucose, glycerol, lactate and acetate were analyzed by high-performance liquid chromatography (HPLC) with an Agilent1200 (Agilent, Co. Ltd USA) equipped with UV absorbance and refractive index detectors (RID) and a Bio-Rad Aminex HPX-87H column (300 × 7.8 mm). The mobile phase was 5 mmol/L H_2_SO_4_, The flow rate was 0.6 mL/min, and the column temperature was 50 °C. Culture broth was centrifuged at 10,000 rpm for 10 min, and 10 μL of the diluted sample was injected into the HPLC instrument.

The analysis of fatty acids and fatty alcohols was performed via HPLC with an Agilent 1200 (Agilent, Co. Ltd. USA) equipped with RID and a SilGreen ODS C18 column (4.6 mm × 250 mm, 5 μm) according to the reported research [34]. The mobile phase was methanol: water: acetic acid (90:9.9:0.1, v/v/v). The column temperature was 26 °C with a flow rate of 1.0 mL/min. Five-milliliter samples of fermentation combined with 500 µL of 10 mol/L HCl were extracted with 2.5 mL of ethyl acetate at 10 °C and 260 rpm for 2 min. The mixtures were shaken vigorously for a few seconds before they were placed in a rotary shaker incubator. After extraction, the mixtures were left static for 10 min and the organic layer was then transferred to a new centrifuge tube. After centrifugation at 12,000 rpm for 5 min, the clear supernatant was collected and filtered through a 0.45-μm millipore filter and injected into the HPLC-RID system for analysis.

### Whole-genome transcriptional analysis

Three replicates of the fatty alcohol-overproducing strain MGKCBA/pL1 and three replicates of the control strain W/pL1 were cultured in modified LB medium as described above. Cells of the two genotypes were mixed separately after 12 h of induction with IPTG and were harvested by quick centrifugation (at 10,000*g*, 4 °C for 1 min) and then immediately frozen in liquid nitrogen. Total RNA was extracted using an RNeasy Mini kit (Qiagen, Valencia, CA, USA) following lysozyme treatment. The total RNA in each sample was quantified and qualified with an Agilent 2100 Bioanalyzer (Agilent Technologies). Pair-end (PE) index libraries were constructed according to the manufacturer’s protocol (NEBNext^®^ Ultra™ Directional RNA Library Prep Kit for Illumina^®^). Sequencing was performed using a 2 × 100 PE configuration. Image analysis and base calling were conducted by the HiSeq Control Software (HCS) + OLB + GAPipeline-1.6 (Illumina) in the HiSeq instrument. The sequences were processed and analyzed by GENEWIZ (Suzhou, China). More information can be found in Additional file 1: Method S1.

## Additional file

10.1186/s12934-016-0524-5 Additional information.

## References

[1] Liao JC, Mi L, Pontrelli S, Luo S. Fuelling the future: microbial engineering for the production of sustainable biofuels. Nat Rev Microbiol. doi:10.1038/nrmicro.2016.3. 10.1038/nrmicro.2016.32 27026253

[2] Dellomonaco C, Fava F, Gonzalez R (2010). The path to next generation biofuels: successes and challenges in the era of synthetic biology. Microb Cell Fact.

[3] Tan X, Yao L, Gao Q, Wang W, Qi F, Lu X (2011). Photosynthesis driven conversion of carbon dioxide to fatty alcohols and hydrocarbons in cyanobacteria. Metab Eng.

[4] Zheng YN, Li LL, Liu Q, Yang JM, Wang XW, Liu W (2012). Optimization of fatty alcohol biosynthesis pathway for selectively enhanced production of C12/14 and C16/18 fatty alcohols in engineered *Escherichia coli*. Microb Cell Fact.

[5] Cai D, Dove J, Nakamura N, Sanders LJ, Klinman JP (2011). Engineered reversal of the β-oxidation cycle for the synthesis of fuels and chemicals. Nature.

[6] Lennen RM, Pfleger BF (2012). Engineering *Escherichia coli* to synthesize free fatty acids. Curr Opin Biotechnol.

[7] Bird AW, Erler A, Fu J, Hériché JK, Maresca M, Zhang Y (2012). High-efficiency counterselection recombineering for site-directed mutagenesis in bacterial artificial chromosomes. Nature Met.

[8] Liu Y, Yang M, Chen J, Yan D, Cheng W, Wang Y (2016). PCR-based seamless genome editing with high efficiency and eidelity in *Escherichia coli*. PLoS ONE.

[9] Andreas S, Rude MA, Xuezhi L, Emanuela P, Cardayre SB (2010). Microbial biosynthesis of alkanes. Science.

[10] Xu P, Gu Q, Wang W, Wong L, Bower AG, Collins CH (2013). Modular optimization of multi-gene pathways for fatty acids production in *E. coli*. Nat Commun.

[11] Desbois AP, Smith VJ (2010). Antibacterial free fatty acids: activities, mechanisms of action and biotechnological potential. Appl Microbiol Biotechnol.

[12] Willis RM, Wahlen BD, Seefeldt LC, Barney BM (2011). Characterization of a fatty acyl-CoA reductase from *Marinobacter aquaeolei* vt8: a bacterial enzyme catalyzing the reduction of fatty acyl-CoA to fatty alcohol. Biochemistry.

[13] Runguphan W, Keasling JD (2014). Metabolic engineering of *Saccharomyces cerevisiae* for production of fatty acid-derived biofuels and chemicals. Metab Eng.

[14] Rupilius W, Ahmad S (2006). The changing world of oleochemicals. Palm Oil Dev.

[15] Hofvander P, Doan TP, Hamberg M (2011). A prokaryotic acyl-CoA reductase performing reduction of fatty acyl-CoA to fatty alcohol. FEBS Lett.

[16] Liu A, Tan X, Lun Y, Lu X (2013). Fatty alcohol production in engineered *E. coli* expressing marinobacter fatty acyl-CoA reductases. Appl Microbiol Biotechnol.

[17] Kalim MA, Turner NJ, Jones PR (2012). Carboxylic acid reductase is a versatile enzyme for the conversion of fatty acids into fuels and chemical commodities. Proc Natl Acad Sci USA.

[18] Steen EJ, Yisheng K, Gregory B, Zhihao H, Andreas S, Amy MC (2010). Microbial production of fatty-acid-derived fuels and chemicals from plant biomass. Nature.

[19] Rowland O, Domergue F (2012). Plant fatty acyl reductases: enzymes generating fatty alcohols for protective layers with potential for industrial applications. Plant Sci.

[20] Haushalter RW, Dan G, Deutsch S, The L, Chavkin TA, Brunner SF (2015). Development of an orthogonal fatty acid biosynthesis system in *E. coli* for oleochemical production. Metab Eng.

[21] Cao YX, Xiao WH, Liu D, Zhang JL, Ding MZ, Yuan YJ (2015). Biosynthesis of odd-chain fatty alcohols in *Escherichia coli*. Metab Eng.

[22] Chan DI, Vogel HJ (2010). Current understanding of fatty acid biosynthesis and the acyl carrier protein. Biochem J.

[23] Seyfzadeh M, Keener J, Nomura M (1993). *SpoT*-dependent accumulation of guanosine tetraphosphate in response to fatty acid starvation in *Escherichia coli*. Proc Natl Acad Sci USA.

[24] Spencer AK, Greenspan AD, Cronan JE (1978). Thioesterases I and II of *Escherichia coli*. Hydrolysis of native acyl-acyl carrier protein thioesters. J Biol Chem.

[25] Youjun F, Cronan JE (2009). A new member of the *Escherichia coli* fad regulon: transcriptional regulation of *fadM* (*ybaW*). J Bacteriol.

[26] Chou KC, Shen HB (2008). Cell-PLoc: a package of web servers for predicting subcellular localization of proteins in various organisms. Nature Protoc.

[27] Baba T, Ara T, Hasegawa M, Takai Y, Okumura Y, Baba M (2006). Construction of *Escherichia coli* K-12 in-frame, single-gene knockout mutants: the Keio collection. Mol Syst Biol.

[28] Glen M, Benson BK, Anne G, Waldrop GL (2009). A tale of two functions: enzymatic activity and translational repression by carboxyltransferase. Nuc Acids Res.

[29] Vaistij FE, Boudreau E, Lemaire SD, Goldschmidt-Clermont M, Rochaix JD (2000). Characterization of Mbb1, a nucleus-encoded tetratricopeptide-like repeat protein required for expression of the chloroplast *psbB/psbT/psbH* gene cluster in *Chlamydomonas reinhardtii*. Proc Natl Acad Sci USA.

[30] James ES, Cronan JE (2004). Expression of two *Escherichia coli* acetyl-CoA carboxylase subunits is autoregulated. J Biol Chem.

[31] Sardessai Y, Bhosle S (2002). Tolerance of bacteria to organic solvents. Res Microbiol.

[32] Zaldivar J, Martinez A, Ingram LO (2000). Effect of alcohol compounds found in hemicellulose hydrolysate on the growth and fermentation of ethanologenic *Escherichia coli*. Biotechnol Bioeng.

[33] Inoue A, Horikoshi K (1989). A *Pseudomonas* thrives in high concentrations of toluene. Nature.

[34] Liu Y, Chen T, Yang M, Wang C, Huo W, Yan D (2014). Analysis of mixtures of fatty acids and fatty alcohols in fermentation broth. J Chromatogr A.

