# Supplementary material for: High production of fatty alcohols in Escherichia coli with fatty acid starvation
Source: Microb Cell Fact. 2016 Jul 27;15:129. doi: 10.1186/s12934-016-0524-5 (PMC4964107; doi:10.1186/s12934-016-0524-5)
Supplement: Supplementary file 1 — 10.1186/s12934-016-0524-5 Additional information. [file 12934_2016_524_MOESM1_ESM.doc]

Supplementary information

**High production of fatty alcohols by metabolically engineered *Escherichia coli* with fatty acid starvation**

Yilan Liu^a^ Sha Chen^b^, Jinjin Chen^c^, Jiemin Zhou^c^, Yanyan Wang ^a^, Maohua Yang^c^ , Xianni Qi^a^, Jianmin Xing^c^, Qinhong Wang^a^, Yanhe Ma^a^

^a^ Tianjin Institute of Industrial Biotechnology, Chinese Academy of Sciences, 32 XiQiDao, Tianjin Airport Economic Area, Tianjin 300308, China

^b^ Institute of Chinese Materia Medica, China Academy of Chinese Medical Sciences, No. 16, Nanxiaojie, Dongzhimennei, Beijing 100700, PR China

^c^ National Key Laboratory of Biochemical Engineering, Institute of Process Engineering, Chinese Academy of Sciences, Beijing 100190, PR China;

Correspondence should be addressed to X.J.M. (jmxing@ipe.ac.cn) or Q.H.W. (wang_qh@tib.cas.cn)

**Table S1**

Results of in-situ separation fermentation.

| Fatty alcohol production (g/L) | With tridecanol | | Without tridecanol | |
| --- | --- | --- | --- | --- |
|  | W/pL1 | MGL2 | W/pL1 | MGL2 |
| Total | 0.49±0.03 | 2.23±0.19 | 0.46±0.02 | 2.01±0.13 |
| Tridecanol layer | 0.49±0.03 | 2.23±0.19 | ----- | ----- |
| Medium | N | N | N | 2.01±0.13 |
| Cell | N | N | 0.46±0.02 | N |

Note: atty alcohol production = mean±SD, N: not detected, -----: not tested.

**Table S2.** Primers used in this study

| **Primers** | **Sequence** |
| --- | --- |
| tesCp1 | CGTAATCTGGCGGTATTAACCCTGTAATTAATTTGCATAGTGGCAATTTTACGTTTTGTGGTGCCGGATGCTCAAGCCGCATCCGGCGACACCCGGAATAGTGACGGAAGATCACTTCGCAG |
| tesCp2 | CATATGGACAGTTTTCCCTTTGATACGTTTTGTGGTGCCGGATGCTCAAGCCGCATCCGGCGACACCCGGAATA |
| tesBp1 | ATCACGCATTTCTGCCTGTAATTAGCCCGTAATTCAGACCATTGCACCCAAAAAATAGCCGGAGGTGAAAACCGTCCGGCTGTTTTTTGCAGTGCTTGTTGTGACGGAAGATCACTTCGCAG |
| tesBp2 | GCATATGGACAGTTTTCCCTTTGATAAAAATAGCCGGAGGTGAAAACCGTCCGGCTGTTTTTTGCAGTGCTTGTT |
| tesAp1 | CCGACGGACTTCTTAAGATGATGAACTTCAACAATGTTTTCCGCTGGCATATGACTCATAAAGCAACGGAGATCCTGACAGGTAAAGTTATGCAAAAATCGTGACGGAAGATCACTTCGCAG |
| tesAp2 | TGCATATGGACAGTTTTCCCTTTGATATGACTCATAAAGCAACGGAGATCCTGACAGGTAAAGTTATGCAAAAATC |
| FARF | CG***GGATCC***ATGGCAATACAGCAGGTAC ATCACG |
| FARR | CG***GAATTC***TCAGGCAGCTTTTTTGCGCTG |
| ptaackAp1 | CTATGGCTCCCTGACGTTTTTTTAGCCACGTATCAATTATAGGTACTTCCGTGACGGAAGATCACTTCGCAGA |
| ptaackAp2 | TTATTTCCGGTTCAGATATCCGCAGCGCAAAGCTGCGGATGATGACGAGAGGAAGTACCTATAATTGATACGTGGCTAAAAAAACGTCAGGGAGCCATAGATCAAAGGGAAAACTGTCCATATGC |
| ldhAp1 | TTAAACCAGTTCGTTCGGGCAGGTTTCGCCTTTTTCCAGATTGCTTAAGTTTTGCAGCGTAGTCTGAGGTGTAGGCTGGAGCTGCTTC |
| ldhAp2 | ATGAAACTCGCCGTTTATAGCACAAAACAGTACGACAAGAAGTACCTGCAACAGGTGAACGAGTCCCATATGAATATCCTCCTTAG |

**Method. S1** Whole-genome transcriptional analysis

Total RNA was extracted using the RNeasy Mini kit (Qiagen, Valencia, CA, USA) following lysozyme treatment. Total RNA of each sample was quantified and qualified by Agilent 2100 Bioanalyzer (Agilent Technologies), and 1 μg total RNA with RIN value above 7 was used for following library preparation. Pair-end index libraries were constructed according to the manufacturer’s protocol (NEBNext® Ultra™ RNA Library Prep Kit for Illumina®). Large ribosomal RNA was depleted from bacteria total RNA using RiboMinus Bacteria Module (Invitrogen) and the ribosomal depleted mRNA was then fragmented, and primed with random primers. First strand cDNA was synthesized using ProtoScript II Reverse Transcriptase and the second-strand cDNA was synthesized using Second Strand Synthesis Enzyme Mix. The double-stranded cDNA purified using AxyPrep Mag PCR Clean-up (Axygen) was then treated with End Prep Enzyme Mix for end repairing, 5’ Phosphorylation and dA-tailing in one reaction, followed by ligation to adaptors with a “T” base overhang.

Size selection of Adaptor-ligated DNA was then performed using AxyPrep Mag PCR Clean-up (Axygen), and fragments of ~400 bp (with the approximate insert size of 250 bp) were recovered. Each sample was then amplified by PCR for 11 cycles using P5 and P7 primers, with both primers carrying sequences which can anneal with flow cell to perform bridge PCR and P7 primer carrying a six-base index allowing for multiplexing. The PCR products were cleaned up using AxyPrep Mag PCR Clean-up (Axygen), validated using an Agilent 2100 Bioanalyzer (Agilent Technologies), and quantified by Qubit and real time PCR (Applied Biosystems). Then libraries with different indexes were multiplexed and loaded on an Illumina HiSeq instrument according to manufacturer’s instructions (Illumina, San Diego, CA, USA). The *Escherichia coli* str. K-12 substr. MG1655 reference genomic DNA and reference gene was download from (<http://www.ncbi.nlm.nih.gov/nuccore/NC_000913.3>). Sequencing was carried out using a 2x100 paired-end (PE) configuration; image analysis and base calling were conducted by the HiSeq Control Software (HCS) + OLB + GAPipeline-1.6 (Illumina) on the HiSeq instrument. Prior to mapping reads to the reference database, we filtered all raw reads to remove adaptor sequences, ambiguity reads (including 10% N base) and filtering reads with low quality by NGS QC Toolkit (v2.3) with parameter cut Off Qual Scorre equaling 30. The remaining reads were aligned to the *E. coli* genome using tophat2 (TopHat v2.0.9, http://ccb.jhu.edu/software/tophat/index.shtml) with bowtie2 (version 2.1.0, http://bowtie-bio.sourceforge.net/bowtie2/index.shtml), allowing up to two base mismatches.

We use the Cufflinks (cufflinks v2.1.0,http://cufflinks.cbcb.umd.edu/) measures transcript abundances in Fragments Per Kilo base of exon per Million fragments mapped (FPKM), either using reference gene. Cufflinks uses a statistical model of paired-end sequencing experiments to derive a likelihood for the abundances of a set of transcripts given a set of fragments. This likelihood function can be shown to have a unique maximum, which Cufflinks finds using a numerical optimization algorithm. The program then multiplies these probabilities to compute the overall likelihood that one would observe the fragments in the experiment, given the proposed abundances on the transcripts.

Differentially expressed sequences between two samples (libraries) were identified by the program cuffdiff (v2.1.1, http://cufflinks.cbcb.umd.edu/). An overview of the Cuffdiff approach to gene-level differential analysis of RNA-seq data as follow: (1) The variability in fragment count for each gene across replicates is modeled; (2) The fragment count for each gene is estimated in each replicate, along with a measure of uncertainty in this estimate arising from ambiguously mapped reads; (3) The algorithm combines estimates of uncertainty and cross-replicate variability under a beta negative binomial model of fragment count variability to estimate count variances for each transcript in each library; (4) These variance estimates are used during statistical testing to report significantly differentially expressed genes and transcripts. Genes with p-value and FDR (q-value) less than 0.05 and expression changing larger than 2 times were identified as significantly differentially expressed. The sequences were processed and analyzed by GENEWIZ.
